# Etiology, Clinical Phenotypes, Epidemiological Correlates, Laboratory Biomarkers and Diagnostic Challenges of Pediatric Viral Meningitis: Descriptive Review

**DOI:** 10.3389/fped.2022.923125

**Published:** 2022-06-16

**Authors:** Saleh M. Al-Qahtani, Ayed A. Shati, Youssef A. Alqahtani, Abdelwahid Saeed Ali

**Affiliations:** ^1^Department of Child Health, College of Medicine, King Khalid University, Abha, Saudi Arabia; ^2^Department of Microbiology and Clinical Parasitology, College of Medicine, King Khalid University, Abha, Saudi Arabia

**Keywords:** children, virus, meningitis, symptoms, epidemiology, diagnosis

## Abstract

Meningitis is an inflammation of the brain and spinal cord meninges caused by infectious and non-infectious agents. Infectious agents causing meningitis include viruses, bacteria, and fungi. Viral meningitis (VM), also termed aseptic meningitis, is caused by some viruses, such as enteroviruses (EVs), herpesviruses, influenza viruses, and arboviruses. However, EVs represent the primary cause of VM. The clinical symptoms of this neurological disorder may rapidly be observed after the onset of the disease, or take prolonged time to develop. The primary clinical manifestations of VM include common flu-like symptoms of headache, photophobia, fever, nuchal rigidity, myalgia, and fatigue. The severity of these symptoms depends on the patient's age; they are more severe among infants and children. The course of infection of VM varies between asymptomatic, mild, critically ill, and fatal disease. Morbidities and mortalities of VM are dependent on the early recognition and treatment of the disease. There were no significant distinctions in the clinical phenotypes and symptoms between VM and meningitis due to other causative agents. To date, the pathophysiological mechanisms of VM are unclear. In this scientific communication, a descriptive review was performed to give an overview of pediatric viral meningitis (PVM). PVM may occasionally result in severe neurological consequences such as mental retardation and death. Clinical examinations, including Kernig's, Brudzinski's, and nuchal rigidity signs, were attempted to determine the clinical course of PVM with various success rates revealed. Some epidemiological correlates of PVM were adequately reviewed and presented in this report. They were seen depending mainly on the causative virus. The abnormal cytological and biochemical features of PVM were also discussed and showed potentials to distinguish PVM from pediatric bacterial meningitis (PBM). The pathological, developmental, behavioral, and neuropsychological complications of PVM were also presented. All the previously utilized techniques for the etiological diagnosis of PVM which include virology, serology, biochemistry, and radiology, were presented and discussed to determine their efficiencies and limitations. Finally, molecular testing, mainly PCR, was introduced and showed 100% sensitivity rates.

## Introduction

Meningitis is a neuropathological disorder due to an inflammation of the meninges that enclose the brain and spinal cord (1). This condition is caused by infectious and non-infectious agents. Non-infectious causes of meningitis include some systemic diseases, drugs, and neoplasms (2). Infectious agents that cause meningitis include viruses, bacteria, and fungi. However, viruses are the most common causative agents of meningitis. Viral meningitis (VM), also called aseptic meningitis, is considered a slow and mild viral disease and regarded as a typical model for chronic nervous system diseases (1, 3). VM is the primary type of aseptic meningitis that commonly affects children. Several viruses were incriminated in the causation of aseptic VM, like enteroviruses, herpesviruses, orthomyxoviruses, arboviruses, and coronaviruses (3, 4). Enteroviruses (EVs) are the most common viruses that cause meningitis among pediatric patients (4, 5). Non-polio EVs were responsible for the highest incidence rates of pediatric VM (PVM) (6, 7). The severe acute respiratory syndrome-coronavirus-2 (SARS-CoV-2), which is responsible for the ongoing COVID-19 pandemic, was also recognized to cause minor cases of PVM (8). It was also reported that the highest incidences, morbidities, mortalities, and disease infectivity rates of PVM were seen among children between 5 and 10 years old (7, 9). Although no seasonal preference for VM was documented but most commonly observed to occur in summer and autumn (1, 7). The epidemiological correlates of PVM were well-described and reviewed in many parts of the world, particularly in the Middle East (ME) region (10–15). These studies indicated that PVM was most commonly occurring among children and young adults (10). They also confirmed that Echovirus 7 (E-7), E-9, E-11, and E-30 constitute the significant viral agents causing PVM (11, 12). Cases of PVM among newborns and infants have also been reported in several studies (7, 11, 14).

The clinical symptoms of PVM were confirmed to be almost the same in their phenotypes and presentations in all cases of the disease, irrespective of the viral etiology (16). Several risk factors associated with PVM, such as age and immunosuppression, were also investigated and confirmed to affect the disease occurrence and severity (1, 6, 7). Brudzinski's signs, Kernig's signs, or nuchal rigidity were employed as physical examinations by clinicians and considered of significant diagnostic values for all etiological types of meningitis including VM (17, 18). Lumbar puncture (LP) for pleocytosis determination or viral detection in the CSF was recommended to confirm the disease (18). Non-specific signs including fever, chills, abdominal pain, nausea, headache, fast breathing, loss of appetite, neck stiffness, and sensitivity to bright light were totally or partially observed among PVM patients (4, 19). Some neurological manifestations like misconception and blurt vision have also been observed in some PVM cases (20). Complications due to PVM are rare to occur, and spontaneous resolution of the disease is not uncommon (21). Clinical signs, disease history, and physical examinations constitute the potential diagnostic tools for PVM. Biochemistry and cytology of cerebrospinal fluid (CSF) were commonly determined and employed as differential diagnostic tests between VM and BM (2). Serological assays for detecting the viral antigens were also used and proved practical and efficient to diagnose VM (4). Polymerase chain reaction (PCR) is the gold standard test for detection of the viral nucleic acid in the CSF of the PVM patients, with the highest sensitivity rates attained (22–24). PCR was also employed to detect the viral nucleic acids during PVM cases in other clinical specimens like blood, throat and nasal swabs, and stools with high sensitivity and specificity levels obtained (25). Other molecular approaches like next-generation sequencing (NGS) were also recently employed to detect viruses in some clinical specimens of PVM (26). Following the systematic search in the literature, we found some publications addressing meningitis due to different etiologies. We also saw plenty of case reports highlighting the various clinical aspects of the disease among children. However, we did not come across a single review article explicitly discussing the PVM as a compiled presentation. Therefore, we would like to address the issue and provide a comprehensive descriptive presentation of the etiology, clinical phenotypes, epidemiology, laboratory abnormalities, complications, and diagnostic challenges of PVM. These elements of PVM are compared to those of PBM and summarized in Table 1.

**Table 1:** Distinctive etiological, clinical, epidemiological, complications, and diagnostic elements of pediatric viral and bacterial meningitis: Comparative presentation.

	**PVM**	**PBM**	**References**
Etiology	The most commonly reported viral causes of PVM include: • Enteroviruses (EVs): mainly CV-A, CV-B, EV-A71, EV-B69, EV-B73, and EV-D68. • HPeV. • Herpesviruses including HSV-1, HSV-2, VZV, EBV, CMV, HHV-6. • Influenza viruses (type A and B). • Arboviruses including WNFV, JEV, YFV, DENV, EEEV, CHIKV, LACV. • Mumps virus. • LMCV.	Several bacterial organisms were known to cause meningitis (septic meningitis) in children, including *Haemophilus influenzae, Streptococcus pneumoniae, Neisseria meningitides, Escherichia coli*, group B *Streptococcus agalactiae, Staphylococcus aureus, Listeria monocytogenes, Mycobacterium tuberculosis*, and *Pasteurella multocida*.	(7, 27–69)
Clinical features	Generally, the clinical phenotypes of PVM and the course of the illness depend on the causative virus. Unless complicated, PVM cases had usually manifested benign or asymptomatic course. Primary non-specific symptoms, including fever, headache, neck stiffness, malaise, anorexia, fatigue, and vomiting, were noted. In critical cases, meningeal signs, including photophobia, myalgia, cough, sore throat, nuchal rigidity, and skin rash, were also observed.	Pediatric BM has been encountered as a more severe and fatal condition than PVM. Antibiotics therapy significantly reduced the infection risk and severity. The characteristic signs of PBM include sudden onset of fever, headache, and meningismus. Among the severe and hospitalized cases, coma, ICP, seizures, shock, and deaths were reported.	(20, 66, 70–72)
Epidemiology	Some factors and correlates for PVM epidemiology were highlighted in many studies. These included; the causative virus, seasonality, age, gender, immune status of the child, infectivity, morbidity, and CFR. Conclusively, PVM was known to have higher infectivity and morbidity rates but fewer mortalities than BM.	The incidence and prevalence rates of BM were noted to peak among young children and the elderly. Formerly, high CFRs of BM were reported. However, antibiotics to treat the disease augmented with the worldwide use of bacterial vaccines (such as *Streptococcus pneumoniae, Neisseria meningitides*, and *Haemophilus influenza*) significantly reduced the incidence and fatalities of BM among pediatric patients, with the peak incidence in young children and the elderly.	(1, 7, 11, 53, 66, 73–85)
Complications	PVM complications were uncommon to occur. However, some short and long-term neurological consequences were reported. Neuropathological disorders (such as shock and seizures), behavioral consequences (such as sensorineural hearing loss), and Neuropsychological complications (such as sleep disturbances) were observed in PVM patients. Other complications, including meningoencephalitis, myocarditis, pericarditis, acute flaccid paralysis, rhombencephalitis, and deaths, were also mentioned in multiple case reports.	Rational and appropriate application of antibiotics and other therapies were known to limit the occurrence of neurological complications among PBM patients. BM was confirmed as the primary cause of global neurological disabilities among neonates. ICP, stroke, and seizures are the most commonly known complications of BM in children.	(27, 30, 31, 85–92)
Diagnosis	Clinical presentations, physical examinations, virus isolation, serological testing, biochemistry findings, and pleocytosis are suggestive. Molecular detection is definitive for the etiological diagnosis of PVM.	Lumbar puncture for bacterial culture was always recommended to diagnose BM. Gram staining and cultures were the essential tools for BM diagnosis. Cells counts and glucose and protein levels were used as differential diagnostic factors with PVM.	(54, 57, 70, 76–78, 93–97)

*CFR, Case fatality rate; CHIKV, Chikungunya virus; CMV, Cytomegalovirus; CV, Coxsackievirus; DENV, Dengue virus; EBV, Epstein Barr virus; EEEV, Eastern equine encephalitis virus; EVs, Enteroviruses; HHV-6, Human herpesvirus 6; HPeV, Human parechovirus; HSV-1,-2, Herpes simplex virus-1,-2; ICP, Intracranial pressure; JEV, Japanese encephalitis virus; LACV, La Crosse virus; LMCV, Lymphocytic choriomeningitis virus; PBM, Pediatric bacterial meningitis; PVM, Pediatric viral meningitis; VZV, Varicella zoster virus; WNFV, West Nile fever virus; YFV, Yellow fever virus*.

## Methods

A comprehensive systematic electronic search in the leading bibliographic platforms for the different scientific elements of PVM was made in this study. The data obtained were collected, registered, and declared in this study regardless of the geographical and seasonal considerations. The search was accomplished through the PubMed, MEDLINE, and Google Scholar databases. The keywords used to search for the information were *meningitis, virus, pediatric, children, infants, epidemiology, symptoms, laboratory, complications*, and *diagnosis*. The articles considered are those published from the ancient times till the year 2022. Inclusion criteria included articles with confirmed positive cases of PVM. The articles published in non-peer-reviewed journals were excluded. Publications related to all age groups of pediatric patients (neonates, newborns, infants, young children, and old children) were considered in the study. The selected articles were carefully read and summarized, and the retrieved data were used in the study description. The essential scientific elements concerning the disease etiology, clinical phenotypes, epidemiology, laboratory abnormalities, complications, and diagnosis were extracted from the selected publications and recorded. Following the compilation of these data, a descriptive presentation was made.

### Definitions

Meninges is an anatomical designation for the three membrane layers that enclose the brain and spinal cord. The name was derived from the Greek word *meninx*, which means membrane. These membranes have two important protective functions; first, they provide direct protection and support to the tissues of the brain and spinal cord; secondly, they are indirectly protecting them by attaching the brain and spinal cord to the skull and vertebral column bones. These meninges were also known to play a pivotal role in the development of the brain and skull during the evolutionary time of the embryonic stage (98–100). Meningitis is an inflammation of these meninges. From an etiological point of view, there are two major types of meningitis; septic meningitis and aseptic meningitis. Septic meningitis, a type of meningitis caused by pus-producing bacteria (101, 102). Aseptic meningitis is the type of meningitis caused by organisms other than bacteria, including viruses (3, 4, 19, 20, 103).

### Etiology

#### Enteroviruses

Enteroviruses (EVs) are groups of non-enveloped RNA viruses belonging to the family *Picornaviridae* and genus *Enterovirus*. The genus Enterovirus includes nine EVs species named Enterovirus A, B, C, D, E, F, G, H, and J (104–107). Each of these EV species consists of multiple serotypes. The serotypes Coxsackievirus A and B (CV-A and CV-B) belonged to the species EV-A and EV-B, respectively (104). CV-A, CV-B, EV-B69, EV-A71, and EV-B73 are the most common EVs causing PVM (27–29). The enterovirus-D68 (EV-D68) was also reported in some studies as a common cause of PVM, with severe and fatal complications described (30, 31). Historically, EVs were firstly known as causative agents of PVM during the early sixties of the last Gregorian century (108). Later, they were confirmed in several epidemiological investigations to constitute the primary cause of VM in children (7, 32–34). In the Middle East region, most of the PVM cases were also attributed to the EVs group of viruses (10–15, 109). Although EVs-induced PVM registered the highest prevalence rates, it was not seen to result in serious complications among children when compared with EVs-induced encephalitis (110). Human parechoviruses (HPeV) is another group of picornaviruses that cause meningitis, particularly among children. HPeV-3 represents the primary type of HPeVs causing PVM (35, 36).

#### Herpesviruses

Out of the known eight human herpesviruses, six of them (HSV-1, HSV-2, VZV, EBV, CMV, and HHV-6) were recognized to cause PVM (37–39). The most peculiar pathogenic feature of HHVs-associated meningitis is that these viruses can remain latent in the human neurons following primary infection and causes meningitis after being reactivated (37). The different clinical and epidemiological features of recurrent meningitis due to the reactivated latent herpesviruses were recently reviewed and published by Rosenburg and Galen (40). HSV-1 was confirmed in some studies to cause only a few mild cases of PVM but high incidences of encephalitis. On the other hand, HSV-2 was observed to cause a higher number of recurrent PVM cases than HSV-1 (111). Other epidemiological studies indicated the reverse trend, which showed that HSV-1 is a predominant causative agent of PVM compared to HSV-2 (112). PVM due to the varicella-zoster virus (VZV) was also reported in many studies as a complication condition following the primary acute disease (41, 113). Compared to HSV-1 and HSV-2, VZV was identified as responsible for scarce cases of PVM characterized by benign clinical manifestations and spontaneous recovery (114). Interestingly, VZV-associated PVM cases were reported even among children who were vaccinated against the disease (115). Epstein Barr virus (EBV) was also documented in many reports as a significant causative agent of PVM (73, 116, 117). Meningitis due to EBV was described as a self-limiting neurological disorder with residual pathological outcomes detected only in meager cases (117). It has also been noted that EBV is more likely to induce a meningoencephalitis condition rather than PVM (118). The cytomegalovirus (CMV) and human herpesvirus 6 (HHV-6) were also mentioned in a few reports as causative agents of PVM (41–43). An infantile meningitis case due to HHV-6 in a premature infant was also recently reported (44). Generally, a consensus from an overwhelming number of reports indicated that introducing molecular techniques, mainly PCR, for detecting the herpesviral DNA in the CFS of patients vastly improved the identification of HHVs as etiological agents of PVM. Another important and hopeful note is that besides the mild and benign nature of HHVs-related PVM, antiviral agents for treating the patients (e.g., acyclovir) are available and effective.

#### Influenza Viruses

Despite the well-known respiratory nature of the diseases caused by human influenza viruses, some neurological disorders like meningitis (45) and meningioencephalitis (119) were also reported on rare occasions as consequences of the respiratory illness. While a laboratory animal model study suggested that the influenza viruses reach the CNS through the olfactory route (46), another tissue culture study showed that they get access to the CNS via the cranial nerve pathway (47). Many case reports and epidemiological investigations for influenza viruses-induced PVM have been published (48–50). Type A influenza viruses are the predominant influenza viruses reported to be associated with PVM cases. Influenza virus subtypes A-H5N1 (46, 47) and A-H1N1 (49) were confirmed to be involved in some PVM cases as CNS manifestations of the infection. Some reports indicating the involvement of influenza virus type B in PVM were also recently published (51, 52). Conclusively, PVM associated with influenza viruses has been known to be characterized by a mild course of infection and benign outcomes observed.

#### Arboviruses

As these are arthropod-borne viruses, the incidence of their PVM was highly correlated with their geographical and seasonal existence (53). The most commonly known arboviruses to cause meningitis are the West Nile Fever virus (WNFV) and the Japanese encephalitis virus (JEV). WNFV was known to arrive at the CNS through the hematogenous pathway after crossing the blood-brain barrier (BBB) (120). While WNFV was reported in the USA primarily as a causative agent of meningitis in adults, it was observed in other countries to cause more cases of pediatric children (54). It was also reported that WNFV is the predominant arbovirus that causes severe PVM leading to serious complications (55). JEV mainly targets the CNS and is noted to trigger the development of many neurological disorders, including meningitis. It was particularly observed endemic in the Southeast Asian region (53). Following laboratory detection of the JEV in the CSF of patients, some researchers in Japan recommended that JEV should not be ruled out in the differential diagnosis of aseptic meningitis (56). Other arboviral infections were also seen in many studies to induce PVM, including yellow fever virus (YFV) (53), Dengue virus (DENV) (57), Murray Valley encephalitis virus, and St. Louis encephalitis virus (53, 93), Eastern equine encephalitis virus (EEEV) (58, 59) and the Chikungunya virus (CHIKV) (60, 61). Although it is a primarily causative virus of encephalitis, the arbovirus La Crosse virus (LACV) was also identified in a large-scale retrospective surveillance study in the USA to cause some cases of PVM (62).

#### Mumps

Before vaccination of children against mumps infection was initiated, it was well known that the mumps virus is the most common virus causing PVM, particularly among non-vaccinated children worldwide (63). The PVM due to mumps infection had considerably declined after introducing the measles-mumps-rubella (MMR) vaccine in the early seventies of the last century (121). Conversely, PVM cases associated with the MMR vaccine were also recently reported in some studies (122, 123). The risk of developing PVM in children vaccinated with either the mumps vaccines, containing different mumps virus strains, or the MMR vaccine was extensively reviewed and reported by Bonnet and co-workers (124). Evaluation of the efficacy of the recently applied combined Measles-Mumps-Rubella-Varicella (MMRV) vaccine and its effect on the development of PVM were also investigated and known to reduce the risk of the disease occurrence (125). Generally, it had been confirmed that the application of these vaccines against mumps greatly changed the frequency of PVM from mumps as the most common causative viral agent of the disease to be supplanted by EVs (125).

#### Other Viruses

The human immunodeficiency virus (HIV) was also known to be associated with some cases of meningitis in adults. The chronic HIV infection results in immunosuppression status and encourages some opportunistic viruses, like CMV, to cause meningitis (123). However, this is becoming of absolute rare occurrence after the advent of the highly active antiretroviral therapy (HAAT). Although no cases of PVM due to HIV were identified, some hospital-based studies indicated that meningitis among HIV-positive children was observed to be associated with other bacterial (126) and fungal (127) organisms. The lymphocytic choriomeningitis virus (LCMV) was also suggested in some studies as a causative virus of PVM without concrete confirmatory evidences obtained up-to-date (64, 65). Rabies virus was also known to induce VM as a complication of the infection but with much lower frequencies (53, 128, 129). Other viral infections like human parvovirus B19 (HPV-B19), Nipah virus, and Bunyaviruses, were also known to be associated with VM in adult cases (53). However, there were no reports of PVM cases caused by these viruses. Some PVM cases were also seen linked to the infection with the severe acute respiratory syndrome-Coronavirus-2 (SARS-CoV-2) (8, 130, 131). On another interesting note, the frequency of the PVM reports due to EVs was known to be markedly declined during the COVID-19 pandemic time in some parts of Europe and Asia (132, 133).

### Clinical Phenotypes

The clinical presentations of PVM and the severity of the disease were found to vary depending on many factors, including the causative virus, child's age, duration of symptoms, and immune status of the patient. The clinical course of PVM ranges from an asymptomatic, mild, critical, and fatal illness. Despite the highest prevalence rates of PVM in many parts of the world, the disease remains benign, self-limiting, and with a better prognosis expected. The non-specific symptoms of PVM include flu-like symptoms like fever, headache, neck stiffness, malaise, anorexia, fatigue, and vomiting (20). Enteroviral PVM is characterized by peculiar clinical features, including the sudden onset of biphasic fever, followed by systemic symptoms and meningeal signs (70). In protracted cases of EV-associated PVM, children may suffer photophobia, myalgia, cough, sore throat, nuchal rigidity, and skin rash (71). The newborns may not manifest overt clinical signs of PVM caused by EVs. However, they may show the signs of other systemic infections like hepatitis, myocarditis, and enteritis (70). Other studies indicated that PVM among newborns can be expressed by overt neurological manifestations like seizures and may lead to death (86). Recently, Alhazmi et al. reported a case of repeated PVM in a 9-day-old baby who presented to the pediatric emergency department (PED). This case was confirmed to be caused by EV in the first episode and HPeV in the second episode. Upon description of the clinical pictures of the disease in the two episodes of this case, different clinical manifestations were observed (24). These findings indicate that the etiological diagnosis of PVM depending on the clinical signs and laboratory indices is unreliable, but molecular analysis for the detection of the causative virus should always be considered. In conclusion, PVM was known to be characterized by benign clinical presentations, spontaneous recovery, and a relatively short hospitalization period. This clinical picture of PVM differs if a concomitant bacterial infection occurs where the patient's condition may deteriorate, and serious complications may occur (134).

#### Kernig's Sign

Kernig's sign approach is a maneuver to diagnose meningitis based on some clinical signs. The technique was invented and reported since a long time by a Russian physician named Vladimir Mikhailovich Kernig. It entitles keeping the patient suspected of meningitis in a sitting or supine position and his/her knees extended to a right angle. If the patient shows resistance or suffers from pain during the extension of the knees, this indicates the spasm of the hamstring muscles and suggests a meningitis condition (135).

#### Brudzinski's Sign

Brudzinski's sign is another type of physical examination used for clinical diagnosis of meningitis. This approach was suggested by the Polish pediatrician Josef Brudzinski in the late years of the nineteen Gregorian century. He described four clinical signs if partially or totally exist then that is suggestive of meningitis. These signs are the cheek sign, symphyseal sign, Brudzinski's reflex, and Brudzinski's neck sign. The detailed application and the reflexes of these signs were adequately described and discussed in some previous reports (136–138). Generally, Brudzinski's sign should be considered positive for meningitis when passive flexion of the patient neck leads to involuntary flexion of the hips and knees (139). In pediatric patients, the reliability of both Kernig's sign and Brudzinski's sign as clinical examinations in diagnosing meningitis was determined and discussed in multiple previous studies (17, 140). Although some studies reported the low sensitivity rates of these signs to diagnose PVM, they still recommended in other studies to be applied (17, 94). We could not see a single published report discussing the application of these examinations in PVM, but they were usually recommended if PBM is suspected. Despite their association with BM, both Kernig's signs and Brudzinski's signs examinations did not constitute differential tools between BM and VM (141).

#### Nuchal Rigidity

Nuchal rigidity is another physical examination utilized by physicians to diagnose meningitis. It was defined as the difficulty in actively forward flexing of the neck secondary to stiff muscles (17). As a possible sign of PVM, nuchal rigidity was confirmed in early studies as a potential tool for diagnosing the disease (88). Moreover, positive nuchal rigidity was promoted as a definite indication for VM, but LP should still be performed when meningeal irritation is suspected (142).

### Epidemiology

#### Age Susceptibility to VM

The susceptibility to VM was seen depending on some risk factors, such as the patient's age, immunity status, and concomitant other viral infections like HIV (1, 53, 93, 103). VM was known to affect the different age groups of people, with more severe clinical consequences detected among pediatric patients than adults (73). Newborns and infants are more likely to develop more acute and critical PVM cases than older children since they are not equipped with mature immune apparatus (7, 11, 74). In a long-term, cohort, population-based study of PVM among hospitalized children in Denmark in 2007, it was confirmed that the first peak of the disease occurred during the first months of the child's life, whereas the second peak of the disease was observed among 5-years old children (7). The authors attributed the peak in the prevalence of PVM during infancy to the lack of passive maternal-specific antibodies and during early childhood to the lack of previous exposure to the causative viruses.

#### Gender Distribution of PVM

Only few epidemiological studies discussed the PVM with reference to the gender distribution. Some reports indicated that males are more likely to develop PVM than females (10, 15, 53, 75). After an intensive search in the literature, we could not see plausible explanations for this type of gender preference. The behavioral and sociocultural factors in the different societies may be involved in PVM acquisition.

#### Seasonality Factor of PVM

PVM was known to occur in all seasons of the year. However, some studies reported higher incidences of the illness in the spring (53), autumn (1, 7, 76), and fall (77, 78). This seasonality in the PVM occurrence was mainly linked to the seasonal distribution of EVs infection, the primary causative factor of PVM (75). It has also been confirmed in some studies that the predominance of the different serotypes of EVs is geographical and seasonal dependent (79, 80). EVs infections were seen to be more prevalent in the summer and fall in the temperate climate region and over the year in the tropical and subtropical regions (20, 79, 81). As a general conclusive remark, we could not judge the factors that control the highest distribution rates of PVM in a particular season or another, and hence there is no seasonal prediction for PVM that can be documented in this report.

#### Infectivity Rates of PVM

Different epidemiological studies reported different infectivity rates of PVM. In a hospital-based a nationwide survey in Northern Finland in 1986, the annual infectivity rate of PVM was estimated as 4.5% out of the total hospitalized children (82). In another recent population-based, retrospective cohort study in South Korea, the infectivity rate of PVM was confirmed to have no pattern and was largely dependent on the prevalence rates of EVs infections (83). The infectivity rates of PVM were ostensibly dependent on the number of cases (sample size) included and the country of the study. We assume that one of the difficulties associated with the determination of infectivity rates of PVM in any epidemiological investigation is the large number of mild cases of the disease which passed unnoticed.

#### Morbidities and Mortalities

Although PVM was documented in many studies characterized by higher infectivity rates PBM, it was known to have lower morbidity and mortality rates (82, 141). The contrast between VM and BM as per the morbidities and mortalities was presented in several previous reports. In an epidemiological study, the mortality rates for PVM and PBM were calculated as 4.5 and 14.5%, respectively (82). The mortality rates of PVM and PBM were also calculated, in a recent national population-based epidemiological study, as 0.002 and 2.0%, respectively (83). Additionally, the morbidities and mortalities of PVM were also estimated in some previous cohort epidemiological studies to be 70 and 10%, respectively (70, 71, 86, 134). Although it had been documented in several studies that the morbidities and mortalities of PVM were dependent on the causative virus, we could not find precise estimates for these correlates in PVM studies caused by the different viral agents. The EV-associated PVM was confirmed to have higher morbidity and mortality rates than other etiological types of PVM (20). The morbidity and mortality rates of PVM were also known as age-dependent. In 2003, some researchers in the USA documented that the PVM represents a fundamental reason for high morbidities, long periods of hospitalizations, and considerable financial losses among infants and young children (84).

### Laboratory Abnormalities

The laboratory investigations were used as a confirmatory diagnostic tool for VM following clinical examination of the suspected patients, as well as they were invested in the differential diagnosis between VM and BM. The laboratory abnormalities associated with VM were particularly described in the literature to indicate the biochemistry and cytology of CSF and patients' blood rather than to indicate the virological, serological, or molecular findings. The major biochemical and cytological pictures associated with PVM and PBM are summarized in Table 2. Examination of the patient's CSF for detection of bacterial growth together with the clinical data served as powerful means to differentiate between the two etiological types of meningitis (2). The seasonal occurrence of VM, especially that due to EVs, is another powerful differential element between VM and BM (77–79). The enormous proportion of neutrophils in the CSF is another characteristic feature of BM (58). The most distinguishing laboratory feature of VM is the lymphocytic pleocytosis in the CSF of the infected individuals (76, 103, 134). In PVM, this type of pleocytosis was accompanied by low glucose levels (2, 54, 57, 76). Although VM is typically characterized by a lymphocytic pleocytosis in the CSF, this may not be observed in young children and neonates (76, 143, 160). It was also observed that the laboratory indices associated with PVM were largely dependent on the causative virus. PVM cases caused by EVs were characterized by neutrophil leukocytosis in the CSF, whereas the decline in the glucose levels in the CSF was mainly detected in the PVM due to mumps virus, HSV-1, and LCMV (76). Moreover, the lymphocyte counts and protein concentrations in the CSF of PVM patients were observed to be significantly different between EVs- and VZV-caused PVM (15, 55). This indicates that the determination of the laboratory abnormalities in the CSF and sera of VM patients is not only helpful to differentiate between VM and BM but also helps to judge the causative virus of PVM. However, the application of the PCR (22, 144) and viral isolation (54, 76) from the CSF of the patient to determine the viral etiology of PVM are of utmost importance for a definite diagnosis. Neutrophilic pleocytosis was also detected in some PVM cases, particularly in the EV-related PVM (161, 162). In addition to these types of pleocytosis, monocyte leukocytosis in the CSF of VM patients was also detected in a hospital-based, large retrospective study conducted in France in 2018 (147). In this study, the CSF monocytes level was noted to be higher among VM than BM patients without significant differences. Conclusively, CSF pleocytosis of all types of cells is a cytological feature of PVM, with some reservations to be used for differential diagnosis between PVM and PBM.

**Table 2:** Cytology and biochemistry of plasma and cerebrospinal fluids of pediatric viral and bacterial meningitis.

	**PVM**	**PBM**	**References**
**Pleocytosis**			
- Total leucocytes counts	During PVM, the CSF-WBCs count was seen to range between 80 and 100 cells/ μl. Although leucocytic pleocytosis may not be detected during some PVM cases, it was also considered a powerful tool to diagnose PVM. The total WBCs counts help in differentiating the different types of meningitis.	Pleocytosis is a common feature of BM. WBCs count of ≥ 500 cells/ μl was indicative of BM.	(2, 76, 93, 103, 134, 143)
- Neutrophilic pleocytosis	Although neutrophilic pleocytosis was detected in some cases during PVM, it was a more prominent feature during BM than PVM.	In PBM cases, exceptionally high levels of the neutrophil count were observed. Neutrophils had been confirmed to account for more than 80% of WBCs in the CSF.	(2, 57, 93, 143, 144)
- Lymphocytic pleocytosis	Lymphocyte count was estimated to be about 80% of the total CSF leucocytes during PVM. Lymphocytic pleocytosis is a prominent laboratory feature during VM.	Lymphocytic pleocytosis had also been reported in many studies as a predominant laboratory feature of BM in pediatric patients. Lymphocytosis was mainly observed during tuberculous meningitis. It was also observed that lymphocytic pleocytosis is more common in neonates suffering from acute BM due to *Streptococcus pneumonia, Neisseria meningitidis*, and *Hemophilus influenzae*.	(2, 14, 54, 57, 143–146)
- Monocytic pleocytosis	Monocyte leukocytosis in the CSF of VM patients was also detected in only a few studies. These studies indicated that monocyte levels slightly increased during VM compared to BM.	A minimal number of studies reported monocytic pleocytosis during PBM. In these studies, there were non-significant elevations of blood monocytes during PBM reported (compared to the healthy and PVM patients).	(147, 148)
**RBCs counts**	The RBC count and ESR were also measured and reported during many cases of PVM. They were observed higher than usual, particularly during herpesviruses meningitis cases in children.	Only a few studies documented the RBCs and ESR levels during PBM. Although ESR was seen to increase during BM, it was not a sensitive biomarker to diagnose BM or differentiate it from VM.	(112, 149, 150)
**Biochemistry**			
- Glucose concentration	In PVM, low glucose levels in the CSF were consistently reported.	Detectable decreased CSF glucose levels during PBM were also observed. It was known to be < 50 mg/dL.	(2, 54, 57, 76, 145)
- Protein concentration	High protein concentrations in the CSF of PVM patients were detected, with the levels varying depending on the causative virus. Elevations in the CRP and other inflammatory proteins were adequately described during some PVM cases.	A remarkable elevation in the protein level was observed during PBM. A concentration of ≥ 1 g/l was deemed as a BM indication. The serum CRP was detected to be substantially elevated during PBM.	(2, 14, 54, 145, 149, 151)
- Lactic acid concentration	The lactic acid concentration was also increased during PVM and recognized as a potential biomarker to differentiate between PVM and PBM.	During BM, lactic acid levels in the CSF were reported and known to exceed 4.2 mmol/l. The CSF lactate concentrations were proved in some studies to be higher in BM than in VM.	(93, 149, 152–154)
- Sodium concentration	Low Na concentrations in the blood of PVM patients were also noted and reported in some studies.	Low initial levels and prolonged decline of Na concentrations during pediatric BM cases were also noted and reported in several studies.	(2, 134, 155)
- Serum amylase levels	One of the most prominent laboratory findings associated with PVM due to Coxsackie viruses is the elevation of the serum amylase levels.	*We did not find any study that reported the serum amylase levels during BM*.	(156, 157)
- Thyroid hormones concentrations	An apparent decline in the thyroid hormones level was detected in the sera of PVM patients, particularly those due to EVs and mumps virus.	Another study also indicated that the thyroid hormones concentrations declined among PBM which is much higher than that observed during PVM.	(158, 159)

*CRP, C-reactive protein; CSF, Cerebrospinal fluid; ESR, Erythrocyte sedimentation rate; BM, Bacterial meningitis; PBM, Pediatric bacterial meningitis; VM, Viral meningitis PVM, Pediatric viral meningitis*.

The qualitative C - reactive protein (CRP) was also detected in the sera of some PVM patients, especially in EVs-PVM cases (2, 86). Recently, the blood biomarkers of the PVM due to SARS-CoV-2 were adequately described by DeKosky et al. (151). They have notably recognized an elevation in some inflammatory protein markers in the CSF and plasma of infected individuals. Low sodium concentrations in the blood of PVM patients were also noted and reported in some studies (2, 134). This condition of hyponatremia was explained by the inappropriate antidiuretic hormone (SIADH) syndrome associated with PVM. The RBC count and erythrocyte sedimentation rate (ESR) were also seen to be higher than usual during HSV-1 and HSV-2 PVM cases (112). Additionally, one of the most prominent laboratory features associated with PVM cases is the significant elevation of the serum amylase levels, especially among those caused by Coxsackie A and B viruses (156) and mumps virus (157). This pointed out a possible impairment in the pancreatic functions during these viral infections. This biochemical property of PVM was not proved to have an absolute or differential diagnostic value. An increase in the lactate concentration among meningitis cases was also demonstrated in multiple studies to be higher in BM than in VM patients (149, 152–154) and can therefore be regarded as a robust biomarker to differentiate between PVM and PBM. An endocrine analysis for the thyroid function during PVM caused by EVs and mumps showed a noticeable decline in the thyroid hormones concentrations, namely TSH, T3, FT3, and T4, in the sera of infected children (158). Another similar study among PBM patients showed almost similar results with more decrease in the thyroid hormones during PBM than in PVM cases (159).

### Complications and Consequences

Although PVM was well known to be characterized by a benign and rapid course of infection and spontaneous recovery, it was also known to have short and long-term neurological consequences in some cases. These consequences are more likely to occur if bacterial co-infection has taken place. Deaths and severe prolonged neurological disorders due to PVM were described in multiple case reports (86, 87). PVM due to EVs was reported to result in significant levels of mortalities and morbidities, particularly among the neonates and immunocompromised children (29–31). Furthermore, it was also observed that the complications of EVs-related PVM were dependent on the subtype of the virus that causes the disease. For example, EV71 and EV68 subtypes were reported to be responsible for the most severe neurological disorder and outcomes among children's populations (27, 30, 31, 163). The most commonly known fatal complications of EVs-PVM include meningoencephalitis, myocarditis, pericarditis, acute flaccid paralysis, and rhombencephalitis (164–166). In the most severe cases of PVM, fatal conditions of unconsciousness, seizure, and shock were also observed (167). In a recent study, it was shown that the PVM due to HPeV can adversely affect the neurodevelopment in young children resulting in gross-motor functional delay (168). In a long-term retrospective hospital-based study, pathological complications of PVM due to herpes viral infections in neonates and infants were determined. They were seen to include meningeal enhancement, infarct, and subdural collections (169). In addition to the previously-mentioned pathological consequences of PVM, some behavioral complications of PVM were also described in several previous reports. Impairment in the language, sensorineural hearing loss, attention deficit, and hyperactivity disorders were noted among some PVM cases (87). Neuropsychological consequences of VM, such as sleep disturbances, were also observed in some cases (89). In a meta-analysis study, the impacts of PVM on the development and intelligence of children were evaluated. This study concluded that children surviving PVM did not had meaningful deleterious outcomes on their IQ and development as compared to those surviving the PBM (170).

After an intensive search in the literature, we can conclude that the occurrence and severity of the complications due to PVM depend on the causative virus and the age and immune status of the child. It was also evident that factors like the prompt diagnosis, responses of some viruses to the antiviral agents, and the introduction of vaccines to immunize children against the viruses declined the possibility of PVM complications, reducing the hospital durations of stay, and limiting the unnecessary use of antibiotics.

### Diagnostic Challenges

In this section of the article, we discuss the indications, contraindications, usefulness, and difficulties associated with the diagnostic techniques of PVM rather than describing them. The preliminary diagnosis of PVM is always made by the physicians via exploring the disease history, focusing on the symptomatology, and applications of the physical examinations. The clinical signs of PVM cases caused by many viral agents are almost similar in their presentations, severity, and frequency of occurrence; hence they were considered unreliable for definite etiological diagnosis. Physical examinations were also employed to diagnose PVM but may not be helpful for differentiation between VM and BM. Kernig's signs and Brudzinski's signs, the bedside physical examinations for meningitis, were routinely used by physicians with high levels of reliability among adult patients, but their validity for the diagnosis of PVM is controversial (17, 94, 95). The major limitation of these physical examinations as a diagnostic tool for PVM is their ability to stretch the inflamed meninges to result in an adequate and detectable irritation (96). Nuchal rigidity signs, as a physical examination test, were also employed by physicians to diagnose PVM, but they did not help to rule in or rule out the disease (18). Jolt accentuation of headache (JAH) is the most recently used type of physical examination to assess the irritation that occurred due to meningitis. JAH was considered positive if the headache from the inflamed meninges increased upon rotating the patient's head horizontally two or three times per second (97, 140). Although reasonable levels of sensitivity and specificity of the JAH to suggest meningitis were reported, it cannot be deemed a reliable test for definitive diagnosis of VM. JAH was recommended in some studies to be used in emergency settings to exclude meningitis (57, 97). Compared with the other physical examinations, the JAH showed better sensitivity and specificity rates for the diagnosis of meningitis (171). Nearly all the reports in the literature addressed the JAH as a diagnostic test among adult meningitis patients. However, only one publication confirmed the effectiveness of the test during PVM cases caused by echovirus 30 (E30) (172). The reliability of the combination of more than one type of these physical examinations was also evaluated in some studies. It had been confirmed that the JAH test could give 100% sensitivity to diagnose meningitis if combined with both Kernig's and Brudzinski's signs (173). Generally, it can be concluded that the classical signs and the physical examination findings of meningitis are not adequate to establish the diagnosis of PVM. As pediatric consultants, two authors of this article believe that these tests for PVM patients can only be looked at as indicators for meningitis and are required for referral to LP for examination of the CSF.

Lumbar puncture had been used for both diagnostic and therapeutic purposes during clinical cases of PVM. For diagnosis, it has been concluded in several studies that LP should always be suggested for diagnosis of pediatric meningitis if the child manifests fever and seizures, particularly if BM is suspected (74, 90, 174). The other important indication of LP is when the patient presents with unconsciousness or altered mental status (175). LP is an invasive procedure thus contraindicated in children, especially if the clinical symptoms and physical examinations provide sufficient information to qualify for an accurate diagnosis of meningitis (90, 174). Some pathological complications of the LP, such as the post-lumbar puncture headache, were also recognized in a myriad of PVM cases (176). When the CSF collected from patients using LP, several laboratory investigations, including bacteriological examinations and biochemistry tests, always carried out purposefully to confirm the infection and to differentiate between BM and VM. These tests include Gram staining and bacterial culture, RBCs counts, WBCs counts, and glucose and protein levels determination. The abnormal cell counts and pathogenic levels of these macromolecules in PVM patients were adequately described and discussed in many previous case reports and review studies (54, 57, 76–78, 93, 97). Bacterial cultures to isolate and identify the causative agent of meningitis, together with the antibiotic sensitivity test, are the proper and applicable diagnostic practice when PBM is suspected (66).

Virus isolation in cell cultures to identify the causative virus of PVM had previously been practiced and discussed in some reports (156, 177, 178). The CSFs were the routinely used samples for virus isolation. However, stool samples were also used especially when EVs infections were suspected. Stool sampling is not an invasive technique, hence considered the most appropriate during the suspicion of PVM (91). The main limitation related to the virus isolation method as a VM diagnostic tool is the poor sensitivity of some cell cultures to propagate certain strains of viruses (177). The sampling time is another significant determinant of the sensitivity of virus isolation as the virus titers in the body fluids may not be to a detectable level. Utilization of serology to identify the viral antigens or the virus-specific antibodies in samples from PVM patients was also made from ancient times up-to-date. The detection of the neutralizing virus-specific IgG and IgM antibodies in the sera of PVM patients was proved valuable for Coxsackie viruses (178). Serology was also used in several epidemiological studies to diagnose PVM due to mumps virus (67), EVs (179, 180), and herpesviruses (181, 182). Despite the usefulness of serological tests to diagnose PVM, false-negative results were obtained particularly in the early phases of the illness (4, 93). Radiological analysis using neuroimaging techniques for clinical cases of PVM was also employed and recommended as a confirmatory diagnosis (67, 183). It had also been documented that neuroimaging was particularly useful during tuberculous PBM (184). Computed tomography (CT) scan also revealed some characteristic clinical outcomes among tuberculous PBM patients, including leptomeningeal enhancement, hydrocephalus, periventricular infarcts, and tuberculoma (183, 184). Generally, neuroimaging was proved of little diagnostic value for PVM in circumstances other than confirmatory purposes because non-specific findings were obtained. However, recently the MRI technique was used to elucidate features that can distinguish meningitis of viral and non-viral etiologies (185).

All of the above-mentioned diagnostic tools for PVM were confirmed to be characterized by relatively poor sensitivity levels and non-specific nature until the different molecular tests were introduced. Undoubtedly, PCR showed superiority over all approaches as the most definitive test to diagnose VM, hence nominated as the gold standard test (22, 93). Reverse-transcription PCR (RT-PCR) to detect the viral nucleic acid in the CSF of PVM patients revealed a 100% sensitivity rate and proved far superior to the virus isolation technique in both EVs- and HPeV-positives cases of PVM (23, 24). Multiplex PCR is now frequently used in clinical and epidemiological investigations to identify the causative virus in PVM patients (162, 186–188). Additionally, approval of some molecular diagnostic kits, such as FilmArray® BioFire® Meningitis/Encephalitis (ME) panel, have significantly reduced the time for detection of the viral etiology of PVM (188, 189). A particular point of concern for the use of PCR during EVs-caused PVM was raised, which indicated that the test is superior to serology as it is significantly helpful for the diagnosticians to differentiate between the various serotypes of EVs causing the disease (76). Conclusively, the PCR is a practical test to identify the exact viral etiology of PVM, which helped to reduce the time of detection, limit the use of unnecessary antimicrobial drugs, and shorten the duration of hospital stay. Other molecular tests, including next-generation sequencing (NGS) and metagenomic next-generation sequencing (mNGS), were also used in numerous studies to diagnose infectious meningitis (26, 190, 191). The usefulness of mNGS enables identifying a wide range of pathogenic organisms in a single assay, including viral, bacterial, fungal, and parasitic causes of meningitis (192, 193). Additionally, the mNGS approach was proved highly useful as it can not only detect the causative virus but can further determine the viral genotype in the clinical specimens (194).

## Conclusions

Following the subtle systematic search in the literature for the various aspects of PVM, the following conclusive points can be highlighted:

- Various viral etiologies for PVM were recognized. However, EVs were the first and most commonly reported causes of PVM. The seasonal occurrence of PVM in specific geographical locations of the globe was found to be associated with the arboviruses' infections. The incidences of PVM due to some viruses were drastically declined after the introduction and application of vaccines.
- PVM cases were more prevalent worldwide but clinically less severe and manifest with benign presentation and spontaneous recovery among children as compared to PBM. They were seen only critical and fatal among newborns, infants, and immunocompromised children.
- As retrieved from the previously published studies, the epidemiological correlates of PVM are mainly dependent on the causative virus and the age of the child.
- The laboratory abnormalities due to PVM were described in plenty of clinical studies. These had mainly focused on the biochemistry and cytology of CSF and blood of PVM patients.
- The pathological, developmental, behavioral, and neuropsychological complications of PVM were adequately reviewed and discussed in this report. Deaths, seizures, shock as well as severe and long-lasting neurological disorders were reported.
- Throughout the history of PVM, the circumstantial, clinical, virological, and molecular tests were utilized as diagnostics to reach the etiology of the disease. However, PCR and the other molecular tests revealed the highest sensitivity rates.

## Author Contributions

All authors listed have made a substantial, direct, and intellectual contribution to the work and approved it for publication.

## Funding

The authors extend their appreciation to the Deanship of Scientific Research at King Khalid University (KKU), Saudi Arabia, for funding this research project; Grant Code R.G.P.1/234/43.

## Conflict of Interest

The authors declare that the research was conducted in the absence of any commercial or financial relationships that could be construed as a potential conflict of interest.

## Publisher's Note

All claims expressed in this article are solely those of the authors and do not necessarily represent those of their affiliated organizations, or those of the publisher, the editors and the reviewers. Any product that may be evaluated in this article, or claim that may be made by its manufacturer, is not guaranteed or endorsed by the publisher.
